# The impact of multiple language exposure on cognition during childhood: evidence from the UK Millennium Cohort Study

**DOI:** 10.3389/fpsyg.2023.1158333

**Published:** 2023-05-18

**Authors:** Jonathan D. Clayden, Steven Hope, Froso Argyri, Sezgi Goksan, Artemis Stefani, Li Wei, Frederique Jeanne Liegeois

**Affiliations:** ^1^Developmental Neurosciences Research and Teaching Department, UCL Great Ormond Street Institute of Child Health, London, United Kingdom; ^2^Population Policy and Practice Research and Teaching Department, UCL Great Ormond Street Institute of Child Health, London, United Kingdom; ^3^Centre for Applied Linguistics, UCL Institute of Education, London, United Kingdom

**Keywords:** language exposure, cohort studies, executive function, Millennium Cohort Study, early cognition, cognitive development

## Abstract

**Introduction:**

Many studies argue that exposure to, and use of, multiple languages in childhood has beneficial effects beyond the linguistic domain, including on executive functions (EFs), although recent evidence remains controversial. EFs encompass abilities necessary for regulating goal-directed behaviours in everyday life and, in children, EFs strongly predict later academic achievement and wellbeing. One theoretical framework distinguishes “hot” EFs, which have a reward or affective component, from “cool” EFs that do not. How exposure to more than one language in early childhood modulates hot and cool EFs in later childhood, alongside other environmental and cognitive factors, remains poorly understood.

**Methods:**

We analysed data from the UK Millennium Cohort Study, a large-scale, nationally representative longitudinal cohort study, which provides information on perinatal and environmental factors (e.g., languages spoken in the home, maternal education) alongside cognitive measures assessed in English. At 3 years, we examined the effect of multiple language exposure on the Bracken school readiness assessment (knowledge of shapes, letters, etc.), and on naming vocabulary. At age 11, we examined the predictors of cool EF, measured with a spatial working memory task; hot EF, measured using a gambling task; and vocabulary, measured using a verbal reasoning task.

**Results:**

Data from 16,134 children were analysed. At age 3, a negative effect of multiple language exposure on school readiness and vocabulary was observed, but the difference was smaller with higher maternal education. At age 11, there was also a negative effect on vocabulary, but smaller than that observed at age 3. There were no direct effects of language exposure on either spatial working memory or gambling scores. For hot EF, the multiple language exposure effects were indirect, mediated by early cognition, and the most significant predictor of gambling strategy was sex. For cool EF, school readiness and vocabulary at age 3 were the strongest predictors.

**Discussion:**

Our findings, based on a UK population sample, highlight the importance of considering socioeconomic status and early-life abilities when interpreting the effects of language environments on hot and cool EFs.

## 1. Introduction

It has been estimated that more than half the world's population is bilingual or multilingual (Grosjean, 2010), and the number of children who do not speak the societal (“majority”) language as their first language has increased in most high-income and middle-income countries. For example, 19.3% of pupils in schools in England −1.6 m in total—were recorded as having a first language other than English in the 2020/21 academic year (Department for Education, 2021).

Different linguistic contexts result in measurable differences in vocabulary acquisition: while bilingual children's pooled vocabulary size across languages is generally not found to be smaller than that of monolinguals, research studies that evaluate only one of their languages show that bilingual children typically score lower than their monolingual peers (Junker and Stockman, 2002; MacLeod et al., 2017). Nevertheless, children with the majority language as a second language generally close the vocabulary gap to their peers after several years of consistent exposure (Collier and Thomas, 2017; Dubiel and Guilfoyle, 2017; Oppenheim et al., 2020). Importantly, the effects of bilingual or multilingual experience in childhood have been associated with changes beyond the linguistic domain, namely in executive functions (EFs; Bialystok et al., 2008; Adesope et al., 2010; Bialystok, 2017), which are higher-level cognitive processes required in goal-directed behaviours and problem-solving (Miyake et al., 2000; Gilbert and Burgess, 2008; Diamond, 2013). This phenomenon, referred to as a “bilingual effect,” is however substantially modulated by factors such as socioeconomic status (SES; Naeem et al., 2018), as is vocabulary development (MacLeod et al., 2017).

Although the magnitudes of the bilingualism effects on EF seem to vary across ages and tasks (Ware et al., 2020; Leivada et al., 2021; see van den Noort et al., 2019, for a review), behavioural studies in children of different ages (Calvo and Bialystok, 2014; Crivello et al., 2016; Blom et al., 2017; Tran et al., 2019) have shown that bilinguals perform better than monolinguals on specific EF tasks (e.g., the Attention Network Task; Yang and Yang, 2016). Other studies have not found a bilingual effect on EFs (Antón et al., 2014; Duñabeitia et al., 2014; Gathercole et al., 2014; Dick et al., 2019), and recent meta-analyses and reviews argue that this effect is negligible and very limited once relevant confounding factors are taken into account, including publication bias (Paap et al., 2015; Lehtonen et al., 2018; Giovannoli et al., 2020; Lowe et al., 2021).

Most research studies investigating the neurocognitive effects of bilingualism are cross-sectional studies, whereas population-based longitudinal studies are relatively rare. In addition, the majority of studies examining bilingual effects during development have focused on small, selected samples matched to monolingual groups based on demographic characteristics, such as IQ and parental SES. These small-scale approaches limit statistical power, increase the risk of selection bias, and raise questions of whether findings can be generalized to a wider population (Leivada et al., 2021). We have limited evidence on how potential cognitive differences between children exposed to a single vs. multiple languages at home unfold during development. Complementary population (cohort) studies therefore have a valuable role to play in addressing these questions and the shortcomings of smaller-scale studies.

There has been increased research interest in the role of emotion and motivation in EF, and studies have investigated the role of EF in various affectively charged situations. In the context of a more affective view of EF, therefore, a conceptual distinction between “cool” and “hot” EFs has been proposed (Zelazo and Müller, 2002; O'Toole et al., 2018). This distinction between EFs is based on the extent to which they are related to emotional/motivational or purely cognitive aspects (Montroy et al., 2019)—but see also Peterson and Welsh (2014). Cool EFs tap into cognitive aspects that require abstract problem-solving, free of an affective load (e.g., traditional working memory tasks). In contrast, hot EFs often refer to tasks that are assessing decision-making using rewards (e.g., digital points, fictional money). While there are several studies investigating cool EFs in children exposed to or using more than one language (e.g., van den Noort et al., 2019), to our knowledge, there is only one instance where hot EF was studied through the context of decision-making which was conducted in monolingual and bilingual children (Enke et al., 2022). The period between preschool and late childhood is of particular interest as it precedes adolescence, a phase of differential developmental trajectories for hot and cool EFs (Poon, 2017), and changes in problem-solving, risk-taking (Casey et al., 2008; Mills et al., 2014; Crone et al., 2016) and decision-making (Blakemore and Robbins, 2012). Little is known about how early exposure to multiple languages and other environmental factors modulate hot and cool EFs in childhood.

In population studies, it is well-established that sociodemographic and perinatal variables have a significant impact on EF and language development. There is extensive evidence that prematurity and low birthweight are associated with EF difficulties throughout development (Aarnoudse-Moens et al., 2009; Mulder et al., 2009; van Houdt et al., 2019). Research additionally shows a persisting negative effect of lower SES and lower maternal education on EF (Hackman et al., 2015; Lawson et al., 2018; Vrantsidis et al., 2020). Similarly, SES impacts language development alongside multiple language exposure (MLE; Reilly et al., 2010), and is considered a modulator of bilingual effects on EF (Calvo and Bialystok, 2014; Naeem et al., 2018). The inter-relation between perinatal and sociodemographic variables, MLE, language and EF is complex during development, so SES and perinatal variables should be considered when approaching the question of how MLE impacts cognitive outcomes.

The Millennium Cohort Study (MCS; Connelly and Platt, 2014; Joshi and Fitzsimons, 2016) is a large, ongoing longitudinal study of around 19,000 children born in the UK between September 2000 and January 2002 (https://cls.ucl.ac.uk/cls-studies/millennium-cohort-study/). A range of cognitive skills have been measured at several time-points (“sweeps”), from infancy to adolescence, alongside extensive social and demographic data, including information on languages spoken at home early in life.

Although the MCS is a multidomain study that was not designed specifically to examine language exposure effects, we sought to examine this extensive dataset in light of the controversial literature on language and cognition. The MCS does not include detailed information on spoken or written language usage, but it offers an opportunity to investigate the likely effects of language exposure, environmental and birth-related factors on early and late cognition in the domains of general ability, vocabulary and EF, in a nationally representative sample of thousands of individuals. Our primary aim was to examine the relationship between MLE and early (age 3) and late (age 11) childhood cognition, focussing on lexical knowledge and general abilities. At age 11, the MCS included one typical cool EF measure, namely spatial working memory (the ability to remember and manipulate information of a visuospatial nature). A measure of predominantly hot EF skills was also available, namely a gambling task where risk-taking and decision-making are assessed in the context of a reward. Secondarily, we aimed to characterise the extent to which these relationships are modulated by sociodemographic and perinatal covariates.

The children's vocabulary skills were only assessed in the majority language (English), which may be a second language for many in the MCS cohort, and no firm information was available on the age of acquisition of languages. We therefore predicted that those with MLE may obtain lower scores in the English vocabulary test than their peers at ages 3 and 11 (Bialystok et al., 2010; Marchman et al., 2010; Scheele et al., 2010; Thordardottir, 2019; Blom et al., 2020), but would have an advantage for working memory at age 11. We did not hypothesize an effect of MLE on the gambling task (hot EF measure) at age 11, as observed by Flouri et al. (2019). We first examined predictors of cognition at ages 3 and 11 in separate cross-sectional analyses. Given the complex interaction between sociodemographic, perinatal and MLE variables during development, we also characterized longitudinal effects using a path analysis.

## 2. Methods

### 2.1. Data

Families were selected for the MCS through Child Benefit records and, to ensure adequate sampling, a disproportionately stratified clustered design was used to over-represent children living in Wales, Scotland and Northern Ireland, disadvantaged areas, and, in England, areas with high proportions of ethnic minority groups (Connelly and Platt, 2014; Joshi and Fitzsimons, 2016). 18,818 infants were enrolled onto the study, but only the 18,295 singletons were included in our analysis. Surveys were carried out in the home with the main carer (usually the mother), by trained interviewers. Each of the main MCS surveys received appropriate ethical approval, and the present secondary analysis was additionally approved by the local ethics committee.

Data from the MCS (Hansen, 2014) were downloaded from the UK Data Service, University of Essex and University of Manchester in May 2017. Data were linked across six survey sweeps, when cohort members (CMs) were approximately nine months, three, five, seven, 11 and 14 years of age. Variables were extracted in five general domains: sociodemographic (CM age at sweep, ethnicity, maternal education, family income), perinatal (gestational age, birth weight), general health (chronic conditions), cognitive (including language, working memory and decision-making tasks; see next section) and data on languages (other than English) spoken at home. Full details can be found in Table 1.

**Table 1:** Variables selected for the present study.

**Category**	**Variable(s)**	**Age at sweep(s)**
Sociodemographic	CM's sex	3, 11 yr
	CM's age (in days) at interview	3 yr
	CM's age last birthday	11 yr
	CM's ethnic group: eight-category classification	3, 11 yr
	OECD equivalised income (imputed)	3, 11 yr
	Highest academic qualification (natural mother, if a respondent)	9 mo
Perinatal	CM's gestation time (in days, estimated)	9 mo
	CM's birth weight (converted to kg where necessary)	9 mo
General health	Long-standing illness/health condition	9 mo, 3, 5, 7, 11, 14 yr
Cognitive	Bracken school readiness composite standard score	3 yr
	British Ability Scales: naming vocabulary *t*-score	3 yr
	British Ability Scales: verbal similarities standard score	11 yr
	CANTAB test scores (spatial working memory; Cambridge gambling task)	11 yr
Language	Language(s) spoken at home	9 mo, 3, 5, 7, 11 yr

CM, cohort member; OECD, Organisation for Economic Co-operation and Development; CANTAB, Cambridge Neuropsychological Test Automated Battery.

Where responses from multiple individuals relating to a single data point were recorded in the dataset, we drew from all respondents to maximise data availability. We prioritised responses from the CM themselves, followed by—in order of priority—the CM's main caregiver (most often the mother), then the partner of the main caregiver, and then a proxy. For example, details about each CM's birth weight were obtained from the main caregiver if available, but in a few cases they were only available from the partner, in which cases these responses were used.

#### 2.1.1. Cognitive measures

The following cognitive measures (Hansen, 2014) were used in the analyses, all administered in English:

- School readiness/knowledge of basic concepts (age 3). The **Bracken School Readiness Assessment** (BSRA-R; Bracken, 2002) appraises the child's knowledge of six concepts, namely colours, letters, numbers and counting, shapes, sizes and comparison skills (see De Almeida Maia et al., 2020, for an evaluation of psychometric properties). The outcome measure is a standard score with a mean of 100 and a standard deviation of 15.
- Vocabulary (age 3). The British Abilities Scale II (BAS II) **naming vocabulary** subtest (Elliot et al., 1996) assesses the child's ability to name pictures of increasing difficulty (expressive vocabulary). The outcome measure is a standard score (i.e., adjusted for age) with a mean of 50 and standard deviation of 10.
- Vocabulary (age 11). The **verbal similarity** subtest of the BAS II assesses verbal knowledge and reasoning. The child is asked to name which class (category) a group of spoken words belong to. The outcome measure is a standard score with a mean of 100 and standard deviation of 15.
- The Cambridge Neuropsychological Test Automated Battery (CANTAB; Cambridge Cognition, 2019) **spatial working memory** task (age 11). Participants have to identify the location of “tokens” hidden under “boxes” on a screen, without returning to the same box location twice. As a result, scores reflect strategy as well as retention and manipulation of visuospatial information, and constitute a measure of cool EF. Higher scores indicate better spatial memory skills.
- The CANTAB **Cambridge gambling task** (age 11). In this computerized gambling task, which constitutes a measure of hot EF, participants are informed of the probability of outcomes before making decisions on “bets”. Seven outcome measures are produced from this task (Cambridge Cognition, 2019).

To reduce the number of CANTAB measures, we carried out a principal components analysis (PCA; see below) which resulted in one component per test that we used as our main outcome measure.

#### 2.1.2. Languages spoken at home

In every MCS sweep, parents were asked about languages spoken in the home, which was used to indicate MLE. For analysis of age 3 data, responses were aggregated across the first two sweeps, covering any exposure up to age 3. At age 11, responses were aggregated from sweeps 3–5, covering exposure after age 3 but up to and including age 11. In each case, where a parent reported use of a non-English home language in any relevant sweep, the CM was considered to have MLE, as exposure to English as the majority language was treated as implicit; otherwise they were classified “without MLE”. We acknowledge this is a relatively crude measure of MLE that does not capture important factors such as age at exposure and active use of a non-English language, but it is the best information available in the cohort. Languages spoken at home encompassed the wide range of languages spoken by families resident in the UK.

#### 2.1.3. Other variables

Table 2 lists the incidence, by sex, of birth prematurity, different language groups and potentially confounding conditions. The latter include developmental and language disorders which may conflate with the effects of MLE on cognitive performance.

**Table 2:** Frequencies (and percentages) of CMs of each sex with relevant perinatal, language and general health characteristics.

**Characteristic**	**Count (%), of girls**	**Count (%), of boys**
**Birth prematurity**		
Term (≥37 wk gestation)	8212 (92.5)	8592 (91.3)
Moderate and late preterm (32–37 wk)	482 (5.4)	580 (6.2)
Very preterm (28–32 wk)	55 (0.6)	79 (0.8)
Extremely preterm (< 28 wk)	18 (0.2)	25 (0.3)
**Home language exposure by age 11**		
Welsh	218 (2.5)	213 (2.3)
Other	1410 (15.9)	1459 (15.5)
**Health conditions**		
Cancer	14 (0.2)	12 (0.1)
Malformation	191 (2.2)	192 (2.0)
Developmental disorder	347 (3.9)	696 (7.4)
Language disorder	292 (3.3)	350 (3.7)

Proportions are expressed in each case as a fraction of those CMs for whom relevant information was available. Conditions are as defined in the main text.

Prematurity was derived from gestational age information, which was available for the majority of CMs. The World Health Organisation thresholds for moderate and late premature (32–37 wk), very premature (28–32 wk) and extremely premature (< 28 wk) babies (March of Dimes et al., 2012) were applied to profile the cohort (Table 2). Continuous gestational age (in days) and birthweight (in kilograms) were used in our statistical analyses.

Maternal education was recorded as the highest academic qualification obtained by the CM's mother: equivalent to GCSE (a public examination taken at age 16), equivalent to A Level (a public examination taken at age 18), a higher qualification (including a degree or diploma) or none. Family income was equivalised using modified OECD scales according to household composition (European Commission et al., 1995).

#### 2.1.4. Sample exclusion criteria

Welsh speakers were excluded from analysis for two reasons: (i) dual-language teaching is available and well-integrated in Wales, and (ii) cognitive testing was conducted in Welsh in some cases. There was therefore the possibility that Welsh was the sole language children were exposed to.

CMs who had been diagnosed with conditions known to impact cognitive development were also excluded. Conditions such as cancer, malformations, developmental disorders and language disorders were identified from data on long-standing health conditions. MCS had its own category labels in some sweeps (for example, “Social or behavioural condition, e.g., associated with autism/ADHD”), and used International Statistical Classification of Diseases and Related Health Problems, 10^th^ Revision (ICD-10; World Health Organization, 2019) codes in others, so a best-effort equivalence was established. Cancer encompassed the terms “cancer” and “tumour,” and ICD-10 codes C00–D48. Malformations included ICD-10 codes Q00–Q99. Developmental disorders included cerebral palsy, epilepsy, learning and memory disorders, other neurological disorders, eating disorders and social problems, encompassing ICD-10 codes F50–F59, F70–F79, F82–F98, G00–G99, and R40–R46. Language disorders included dyslexia, speech, hearing and vision problems, encompassing ICD-10 codes F80–F81, H53–H54, H90–H91, and R47–R49.

### 2.2. Principal components analysis

The Bracken school readiness score and BAS II provide overall standard scores for each CM, but the CANTAB produces a series of scores measuring different aspects of its constituent tasks. There are 14 variables for the spatial working memory (SWM) task, and seven for the Cambridge gambling task (CGT). Each constituent variable was transformed using the Yeo–Johnson transformation (Yeo and Johnson, 2000)—a variant of Box–Cox which handles negative raw values—with parameter optimised to minimise transformed absolute skewness, then scaled to have zero mean and unit variance. Extreme outliers with standardised multivariate Mahalanobis distance of greater than 5.0 were then excluded, and the scaling repeated without them until no further outliers remained. Standard principal components analysis was then applied, reducing the dimensionality of the scaled data to just the first principal component for each task (viz. SWM PC1 and CGT PC1) for subsequent analysis. This and all subsequent data analysis was performed with R v4.1.0 (R Core Team, 2021).

### 2.3. Cross-sectional analysis

Regression analyses were used to estimate cross-sectional relationships between MLE status and cognition. Given the large sample size, an indicative significance threshold of α = 0.01 was applied to all of our statistical findings, and confidence intervals (CIs) were calculated at the 99% level.

At age 3, naming vocabulary and school readiness scores were the dependent variables, with CM age at interview, sex, gestational age, birthweight, and household income as predictors. Given the evidence that the effects of MLE on cognitive abilities and vocabulary development are modulated by SES (MacLeod et al., 2017; Naeem et al., 2018), we included interaction terms between MLE status and maternal education, as well as main effect terms for each variable.

At age 11, dependent variables were the verbal similarity scores and scores from the first principal component of each of the Cambridge gambling and spatial working memory tasks (corresponding to hot EF and cool EF, respectively). Predictors included CM age at interview, sex, household income at age 11, maternal education, MLE status and the interaction between maternal education and MLE status.

### 2.4. Path analysis

Path analysis was performed using the *lavaan* R package (Rosseel, 2012), incorporating terms from the age 3 and age 11 sweeps to consider how early life experiences influence later performance. The model was constructed to capture the influence of MLE, SES, age and sex on cognitive scores within each sweep, as well as longitudinal effects of perinatal variables on the age 3 sweep, and age 3 variables on related age 11 variables. The prevalence of missing data in relevant variables varied from 0% (for sex) to 36% (for the CGT scores), and hence a full-information maximum likelihood approach was taken to fit the model. This is a statistically efficient alternative to imputation in the context of structural equation models (Enders and Bandalos, 2001), allowing us to make use of all available data.

## 3. Results

Of the 18,295 singleton CMs in the MCS, 431 were identified as Welsh speakers and 1778 had evidence of a developmental or language disorder, cancer or malformation (see Table 2), and so were excluded from further analysis. The analytic sample comprised 16,134 CMs.

### 3.1. Principal components analysis

For the SWM task (cool EF), 9133 CMs contributed data to the principal components analysis. Two variables—“Double errors 4 boxes” and “Within errors 4 boxes”—showed no variance at all, but the first principal component captured 36% of the variance in the remaining 12 variables. The component was negatively weighted on all of the constituent variables, such that increasing scores reflected faster performance with fewer errors.

For the CGT (hot EF), 9992 CMs contributed data, all seven variables were used, and the first principal component captured 35% of their variance. This component was weighted negatively for test duration, deliberation time and risk adjustment, and positively for the other variables, such that increasing scores reflected a less deliberative and higher-risk approach.

### 3.2. Cross-sectional analysis

**Figure 1:**
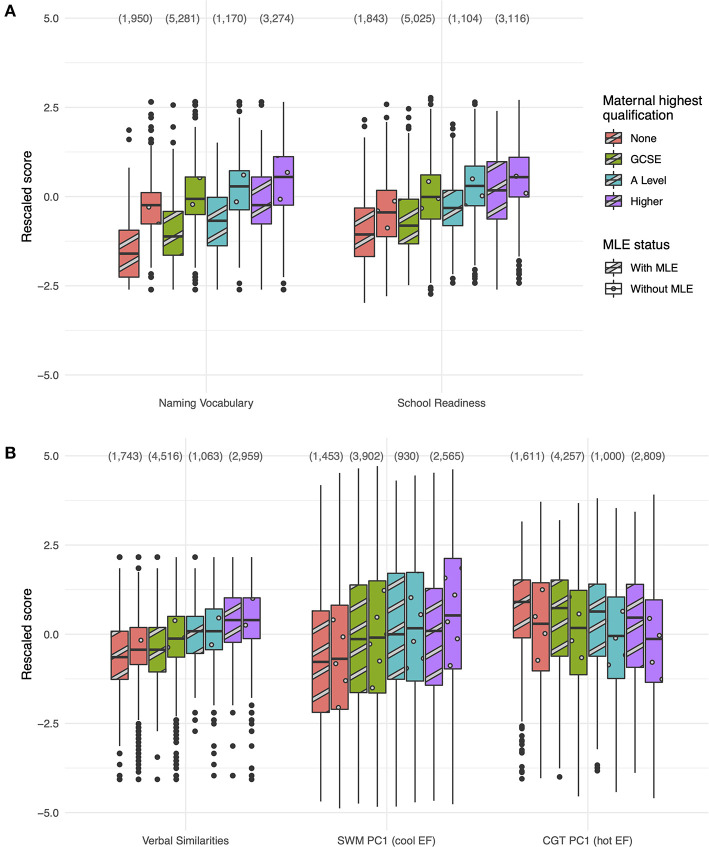
Boxplots of test scores from each of the cognitive tests taken at age 3 **(A)** and age 11 **(B)** according to maternal education and MLE status, after rescaling to have zero mean and unit variance. In each case data are grouped according to MLE status, based on evidence of a non-English language spoken in the home (if any), and the type of the highest academic qualification held by the CM's natural mother. Aggregated counts at each maternal education level are given in parentheses above each plot. MLE, multiple language exposure; SWM, spatial working memory (cool EF); PC, principal component; EF, executive function; CGT, Cambridge gambling task (hot EF).

Boxplots of the cognitive test scores are shown in Figure 1, split by maternal education level and MLE status. Regression results are shown in Table 3.

**Table 3:** Effect sizes (standardised β) for all cross-sectional regression models with cognitive outcomes as dependent variables.

	**3 yr**		**11 yr**		
**Predictor**	**Naming vocabulary**	**School readiness**	**Verbal similarities**	**SWM PC1**	**CGT PC1**
MLE	**−0.392**	**−0.156**	**−0.060**	0.006	**0.099**
Mat. Ed. GCSE	**0.145**	**0.148**	**0.098**	**0.087**	−0.030
Mat. Ed. A Level	**0.133**	**0.154**	**0.110**	**0.078**	**−0.043**
Mat. Ed. Higher	**0.246**	**0.284**	**0.223**	**0.162**	**−0.069**
MLE × Mat. Ed. GCSE	**0.043**	−0.008	−0.007	0.009	−0.016
MLE × Mat. Ed. A Level	**0.041**	0.002	0.017	0.002	< 0.001
MLE × Mat. Ed. Higher	**0.093**	**0.033**	**0.041**	−0.036	−0.014
Male sex	**−0.120**	**−0.114**	**0.054**	**−0.043**	**0.243**
Age	**0.029**	**0.053**	**−0.091**	**0.053**	−0.006
Income	**0.134**	**0.232**	**0.145**	**0.111**	**−0.048**
Gestational age	0.002	**0.036**	–	–	–
Birth weight	**0.056**	0.010	–	–	–

Age and income are taken from the same data sweep as the outcome variable in each case. Effects significant at *p* < 0.01 are shown in bold. MLE, multiple language exposure; SWM, spatial working memory (cool EF); PC, principal component; CGT, Cambridge gambling task (hot EF).

At age 3, two clear trends are observed across the tests (Figure 1A). Firstly, higher maternal education was associated with higher test scores. The advantage of GCSE-level maternal education to CM performance on the naming vocabulary test, relative to no formal qualifications, is highly significant (standardised β = 0.145, 99% CI 0.111 to 0.180); the effect was similar for A Level equivalent qualifications (β = 0.134) and rose substantially for higher education (β = 0.246). For the school readiness test the equivalent β values were 0.148, 0.154 and 0.284, respectively, and each was highly significant. Secondly, CMs with MLE performed less well than those without (main effect β = −0.392 for naming vocabulary, with 99% CI −0.431 to −0.353, and −0.156 for school readiness, with 99% CI −0.197 to −0.116). The interaction between maternal education and MLE was also significant for the naming vocabulary test, with a smaller difference between those with and without MLE at higher education levels. For school readiness a comparable reduced difference was only observed for CMs whose mothers had a higher education (β = 0.033, 99% CI 0.001 to 0.065). 24% of the variance in naming vocabulary and 21% of the variance in school readiness was explained by the predictors.

At age 11, patterns varied by test (Figure 1B). For the verbal similarities test, a benefit of higher maternal education was again observed (β = 0.098 for GCSE versus no formal education, rising to 0.223 for higher education), as was a negative MLE effect on performance, albeit a weaker one (β = −0.060, 99% CI −0.102 to −0.019). Only the interaction between MLE and higher maternal education was significant, such that the MLE effect was effectively cancelled out in this group (β = 0.041, 99% CI 0.008 to 0.073). Spatial working memory (cool EF) scores were significantly and positively associated with maternal education, but not with MLE status. For the gambling task (hot EF), CGT scores were weakly associated with maternal education (β = −0.043 for A Level and β = −0.069 for higher education), and scores were higher for CMs with MLE than those without (β = 0.099, 99% CI 0.055 to 0.142). Less than 10% of the total variance in SWM and CGT scores was explained by the predictors, however.

We examined the associations between the remaining sociodemographic and perinatal variables and each set of cognitive test scores (Table 3). Boys performed worse than girls in both tests at age 3, but the picture was mixed at age 11, with boys performing slightly better in the verbal similarities test and slightly worse in SWM. There was a substantial score difference (reflecting differing strategies) in the CGT. Household income was strongly associated with scores on all tests. The three remaining factors, age in days (at age 3), gestational age and birth weight, only showed weak effects on cognitive test scores.

### 3.3. Path analysis

**Figure 2:**
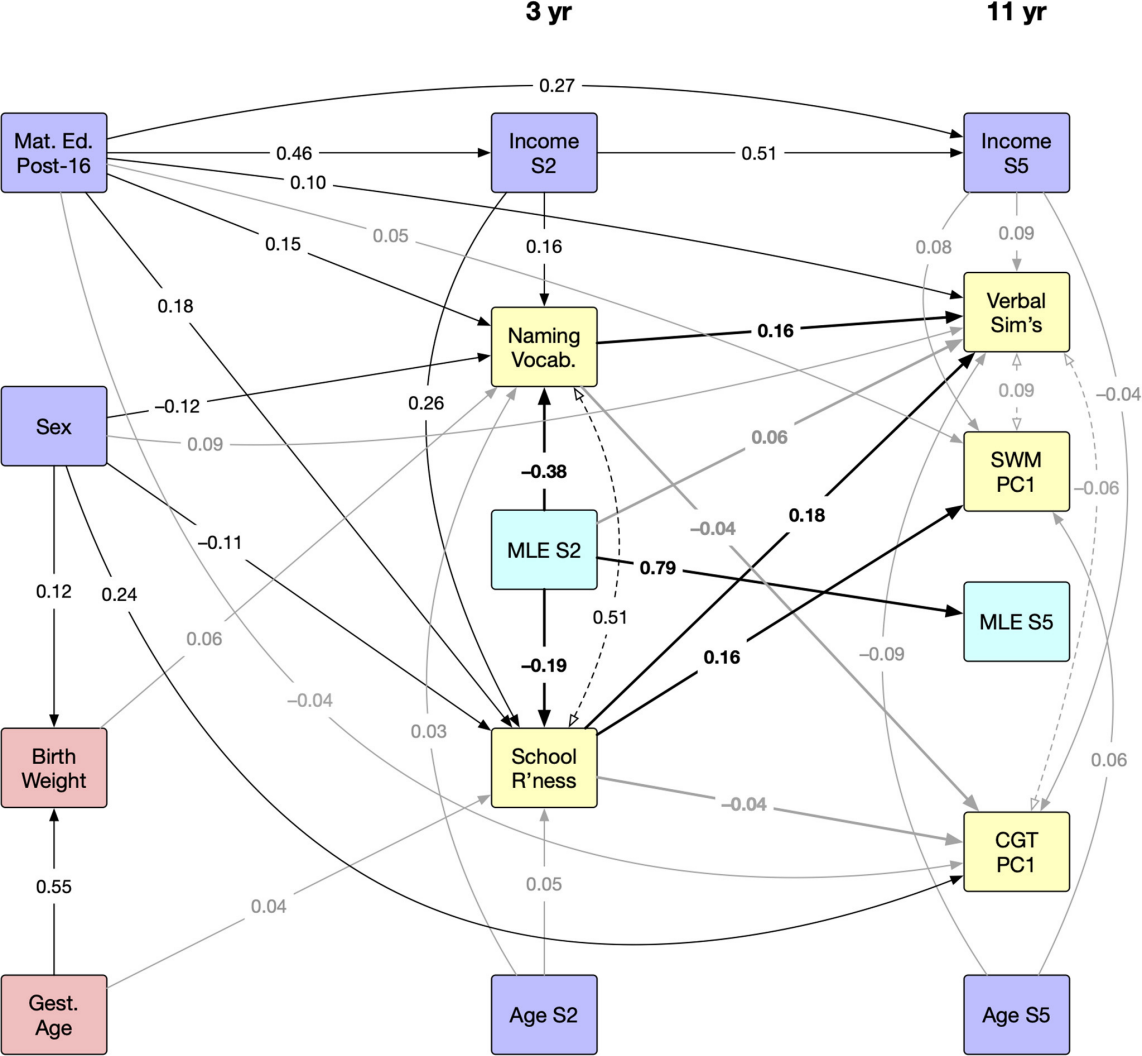
Path analysis diagram showing relationships between sociodemographic (blue), perinatal (red), cognitive (yellow) and language (cyan) variables in the Millennium Cohort Study data. Variables shown in the middle column relate to sweep 2 (age 3), and in the right column to sweep 5 (age 11). Paths with standardised coefficients are shown for every relationship significant at *p* < 0.01, although weaker effects (absolute coefficient below 0.1) are shown in grey. Relationships between cognitive and language variables are highlighted with bold font and heavier lines. Residual covariance terms between cognitive variables are shown with dashed lines and open arrowheads. MLE, multiple language exposure; S2, sweep 2; S5, sweep 5; SWM, spatial working memory (cool EF); PC, principal component; CGT, Cambridge gambling task (hot EF).

Figure 2 shows the results of the path analysis for relationships between MLE, cognitive test scores and covariates, with only terms significant at *p* < 0.01 shown. The maximum likelihood model was found to be an acceptable fit to the available data, with standardised root mean square residual of 0.046 and root mean square error of approximation of 0.055 (Hu and Bentler, 1999).

The key findings from the cross-sectional analyses are observed within each sweep, notably the strong negative MLE effect on cognitive test scores at age 3, and the positive effects of higher maternal education. Longitudinally, there is a positive direct link between early MLE and verbal similarities scores at 11, with no significant additional effect of changed MLE status by the time of the test. Moreover, school readiness and vocabulary at age 3 are more strongly associated with verbal similarities and spatial working memory task performance at age 11 than any variable recorded at age 11. In other words, pre-school cognitive performance is a strong indicator of performance in the period during which children transition from primary to secondary education, while sex is the strongest predictor of gambling strategy. However, we found no direct effect of MLE status (in either sweep) on either spatial working memory or gambling scores.

## 4. Discussion

This analysis of a large, nationally representative cohort study confirmed that the effects of MLE and maternal education on crystallised knowledge interact, and revealed no direct effect of MLE on hot or cool EFs in childhood. At age 3, exposure to a non-English language at home—assumed to be in addition to some English exposure in the absence of detailed information—was associated with lower vocabulary and school readiness scores assessed in English at the same age. At age 11, the effect of MLE on verbal knowledge remained negative, but more weakly so, being more directly linked to pre-academic abilities at age 3. Our study also revealed distinct paths to hot and cool EF performance at age 11. Spatial working memory performance was most strongly associated with maternal education in a cross-sectional analysis, and with earlier knowledge (school readiness and vocabulary at age 3) in a longitudinal analysis. On the other hand, there was a significant effect of MLE on the gambling task results when considered cross-sectionally. However, in a longitudinal analysis, no direct effect of MLE was observed. We suggest that this effect may be subsumed by the indirect effects (e.g., via age 3 scores). SES played a major interacting role at ages 3 and 11 years.

### 4.1. MLE effects at age 3

Although children with MLE in this cohort cannot be reliably described as “bilinguals,” our findings at age 3 accord with previous studies reporting that bilinguals score lower than monolinguals on pre-school measures of receptive vocabulary (Allman, 2005; Bialystok, 2009; Bialystok et al., 2010; Smithson et al., 2014). Lower scores in school readiness are however at odds with results from another population study indicating positive bilingualism effects (Guhn et al., 2016). The Bracken test of school readiness used in the MCS assesses knowledge of letters, numbers, shapes and other concepts in English. Scores are therefore highly influenced by both cognition and knowledge of English (see Snow, 2006; for a review of different school readiness assessments), and do not reflect personal, social or emotional skills.

In the MCS no data were available on age of onset of exposure to English, amount of input children received in English, or knowledge of letters or numbers in the non-English home language. It was therefore not possible to firmly categorise CMs as monolingual, bilingual or multilingual. Other research has suggested that vocabulary differences between bilingual and monolingual children, and between different bilingual groups, can be substantially explained by the language of assessment (MacLeod et al., 2017). Specifically, previous studies have showed that children who are exposed to the majority language after birth had smaller vocabulary size than monolinguals when assessed in the majority language (Oller et al., 2007). In contrast, research shows that bilinguals' vocabulary size can be monolingual-like when children are assessed in their dominant language (Thordardottir et al., 2006; MacLeod et al., 2017). It is therefore likely that lower performance in the assessments of children with MLE at age 3 can be explained by late age of onset of (or sequential) exposure to English, by limited concurrent exposure to English, or by both. This is supported by the relatively strong residual correlation between school readiness and naming vocabulary, visible in Figure 2, which indicates a significant link between these scores that isn't explained by other predictors in the model, and is potentially attributable to the common factor of English proficiency.

Our findings showed positive effects of higher maternal education on the cognitive test variables. Maternal education is a well-known strong predictor of cognitive development in population studies (Bornstein et al., 2013), and so it is not surprising to see its moderating effects here at age 3. Previous research has identified maternal education as a significant predictor of high vocabulary scores in bilinguals (Place and Hoff, 2016; MacLeod et al., 2017). This moderating effect may be due to a range of reasons, including richer and higher language exposure to English and non-English language(s) at home, access to books in English, or more interaction in English with parents and caregivers (Kim et al., 2014; Sorenson Duncan and Paradis, 2018). In bilinguals, it is worth noting that the positive effects of maternal education may be language-specific (Hoff et al., 2018), whereby the mother's language of education impacts positively on the child's vocabulary in that language. In a similar vein, Sorenson Duncan and Paradis (2018) found that mothers with higher levels of education had higher second language fluency and were more likely to use that language with their kindergarten children. We are unable to examine this point directly, however, as there is no MCS data on maternal language of education.

Overall, our findings at age 3 demonstrate an interaction between maternal education and MLE on pre-academic abilities, as measured by vocabulary and school readiness assessments in English. We also found negative effects of younger gestational age and lower weight at birth, confirming the extensive literature reporting vocabulary reductions in children born preterm (Barre et al., 2011; Van Noort-Van Der Spek et al., 2011). More extensive information on maternal cognition, mother-child interactions, child knowledge in the home language, and the child's broader language environment would be required to examine the relative contributions of environmental and genetic factors on the measures used here.

### 4.2. MLE effects at age 11

At age 11, the lower performance found in CMs with MLE at age 3 on crystallised knowledge (measured with verbal reasoning) was substantially mitigated by a direct positive longitudinal effect. This reduced gap in verbal reasoning scores is likely due to the children's extended exposure to English in a range of environments, including at home, at school, and via various media. The “levelling” effect of high maternal education seen at age 3 was replicated, although less strongly, such that children of mothers with a higher education qualification with and without MLE no longer differed in their verbal reasoning abilities at that age. Previous studies have reported that majority (English) language exposure in school, and richer environments outside school, are significant predictors of children's development in that language (Paradis et al., 2017). A more recent study confirmed this point, showing that bilingual children who had been in school longer exhibited higher productive abilities when completing a storytelling task in the majority language (Sorenson Duncan and Paradis, 2018). Altogether, increased exposure to the majority language boosts majority language skills, as formal schooling allows bilingual children to learn and use new vocabulary in various settings. Our study supports this hypothesis in children with MLE from a national cohort.

We found no evidence of a direct MLE effect on either hot or cool EFs at age 11, consistent with a meta-analysis which concluded that bilingualism effects were negligible once other factors were taken into account (Lowe et al., 2021). Our data however revealed distinct early predictors for hot and cool EFs. Our cool EF measure (spatial working memory) was influenced by school readiness scores at age 3, which are highly correlated with English vocabulary scores at that age. This finding is consistent with those from a study at school age (6–9 years) where verbal working memory was predicted by vocabulary and SES, as well as processing speed (Lensing and Elsner, 2018). Overall, our findings suggest that cool EFs in pre-adolescence are better predicted by earlier cognitive measures than by MLE status.

Findings from hot EF (gambling) scores supported our hypothesis of a negligible direct effect of MLE, once longitudinal cognitive factors were taken into account. When examining our principal component from the gambling task, where higher scores reflect a higher-risk strategy preference rather than better performance, sex was the most significant predictor. This finding is consistent with another study in middle childhood, where sex was the only significant predictor of performance on another gambling task (Lensing and Elsner, 2018). Higher risk-taking for boys has been recently reported in the MCS cohort at both age 11 and 14 (Lewis et al., 2021). We only observed indirect effects of MLE longitudinally, via school readiness and vocabulary measured at age 3. Overall, our EF findings confirm distinct predictors for cool and hot EF, in line with theoretical models (Zelazo and Carlson, 2012) and evidence of their distinct developmental trajectories (Lensing and Elsner, 2018).

### 4.3. Strengths and limitations

This study's key strength, compared to many previous studies of EFs and bilingualism or language exposure, is its use of a large and nationally representative cohort, including over 16,000 individuals in our core analyses with information collected on a rich set of birth-related and socioeconomic factors. However, since the data were not gathered with our specific research questions in mind, we had access to only a relatively crude measure of MLE, a trade-off that can be seen as complementary to more specialised and detailed studies on smaller groups. A particular limitation is the lack of a definitive question in the surveys about each child's abilities in the majority language or any others—with our analysis required to infer MLE and possible bilingualism from data about languages spoken at home—or any details of the length or depth of their exposure in spoken or written forms. Moreover, all tests were conducted in English, putting children with English as an additional language at an inherent disadvantage. Nevertheless, the interactions we observed with maternal education re-emphasise, for future studies of bilingualism, language exposure and cognition, that a child's wider intellectual environment is key for fully interpreting any claimed effects, and that groups homogeneous in SES may offer only a limited picture.

Overall, our data showed little effect of MLE in general on hot and cool executive functions in pre-adolescence, once other factors were taken into account. However, our large-scale longitudinal study revealed that their antecedents differ, in line with their related but independent developmental trajectories (Fernández García et al., 2021). Our longitudinal analysis demonstrated that proximate external factors such as maternal education, household income and the linguistic environment strongly influence a child's early cognition, but the impact of these factors wanes over time, with children's cognitive performance affected by a wider range of variables, reflecting the gradually increasing importance of influences beyond the home. Future focussed studies, incorporating richer linguistic data, will be necessary to confirm these findings.

## Data availability statement

Publicly available datasets were analyzed in this study. This data can be found here: UK Data Service (first survey DOI 10.5255/UKDA-SN-4683-1, second survey 10.5255/UKDA-SN-5350-3, third survey 10.5255/UKDA-SN-5795-3, fourth survey 10.5255/UKDA-SN-6411-6, fifth survey 10.5255/UKDA-SN-7464-2, and sixth survey 10.5255/UKDA-SN-8156-2).

## Ethics statement

The studies involving human participants were reviewed and approved by UCL Research Ethics Committee. Written informed consent to participate in this study was provided by the participants' legal guardian/next of kin.

## Author contributions

JC, SH, FA, SG, LW, and FL designed and planned the research. JC and SH acquired, processed, and analysed the data. All authors contributed to interpretation and contextualisation of the findings. JC, SH, FA, AS, and FL wrote the paper. All authors helped refine the paper and approved the final version.
